# A Qualitative Study of Unplanned Hospital Readmissions: Patient Perspectives on Their Hospital to Home Transition

**DOI:** 10.3390/nursrep15060192

**Published:** 2025-05-29

**Authors:** Dale Yeatts, Chetan Tiwari, Samuel Coleman, Michelle Yeatts, Katherine Sobering

**Affiliations:** 1 Department of Sociology, University of North Texas, Denton, TX 76203, USA; samuel.coleman@unt.edu (S.C.); katherine.sobering@unt.edu (K.S.); 2 Department of Geosciences, Georgia State University, 25 Park Place, Atlanta, GA 30302, USA; ctiwari@gsu.edu; 3 Medical City Healthcare, 13155 Noel Rd Suite 2000, Dallas, TX 75240, USA; myeatts1@gmail.com

**Keywords:** nursing, access to health care, hospital discharge planning, social supports, personal stress, neighborhood environment, living conditions

## Abstract

**Background:** Roughly 18% of all patients discharged from hospitals in the United States experience an unplanned hospital readmission (UHR) within 30 days of discharge. This can be life-threatening for patients and costs the U.S. health care system billions of dollars. The Centers for Medicare and Medicaid Services is seeking continued research to identify factors contributing to UHR. Research has viewed the transition from hospital to home in three stages: the pre-discharge stage where the patient is being diagnosed and treated in the hospital, the bridging stage where the patient is being prepared for discharge, and the post-discharge stage where the patient is recovering at home. **Objectives:** Our aims were: (1) to identify factors perceived by patients to influence their recovery during at least one of the three stages of the hospital to home transition and (2) to identify factors perceived by patients as important across all three stages of the transition. **Methods:** To accomplish this, we analyzed information obtained from in-depth, home interviews with 62 participants who had been discharged from a regional hospital roughly 30 days prior to the interview. Our analysis included open-ended readings and the use of qualitative analysis software. **Results:** Factors reported to influence recovery at the pre-discharge stage include appropriate diagnosis, treatment, and financial resources. Factors at the bridging stage include access to health information and social supports. Factors perceived to influence recovery at post-discharge include personal characteristics, social supports, and the environment. **Conclusions:** Participants identified factors at the pre-discharge, bridging, and post-discharge stages believed to be influencing their ability to recover from a hospital stay. Four of these factors were perceived to influence their recovery across multiple stages of the hospital to home transition. These included financial resources, social supports, access to health services, and personal stress.

## 1. Introduction

Unplanned hospital readmissions (UHRs) refer to patients who were discharged from the hospital and then experienced an unplanned readmission within 30 days of their discharge. Roughly 18% of all patients discharged from hospitals in the United States experience an UHR within 30 days of discharge. This can be life-threatening for patients and costs the U.S. health care system billions of dollars [1,2,3]. In many cases, UHRs are unavoidable, particularly where there are comorbidities and progression in the natural history of the patient’s underlying disease or health condition [1]. However, a review of 34 studies, most based on retrospective medical chart reviews, found preventable UHRs to range from as low as 5% to as high as 79% [4,5,6].

In response to this on-going problem, the Centers for Medicare and Medicaid Services (CMS) established the Hospital Readmission Reduction Program (HRRP). Prior to this program, hospitals had a financial incentive to discharge patients as quickly as possible which appears to have contributed, at least in part, to high UHRs [7]. However, this has changed now that HRRP deducts up to 3% of a hospital’s Medicare payments if the hospital’s thirty-day readmission rates are deemed significantly higher than other similar hospitals in their region [8].

Why the high number of UHRs? Hospital managers are seeking answers to this question in order to avoid penalties as well as to successfully treat their patients. CMS is seeking answers as an advocate for older Americans as well as a means of controlling costs. Consequently, research is on-going to determine what factors are contributing to UHRs. In this regard, many studies have relied on large data sets such as that provided by the Centers for Medicare and Medicaid Services (CMS), other census data, and data from hospitals that provide health, demographic, and economic information about their patients [9,10]. This research has been valuable in identifying demographic and economic factors of importance, but it lacks in-depth explanations for UHRs [2]. While there are valuable qualitative studies that have been conducted for this purpose, see for example [11,12,13,14], few of these consider the patient’s entire transition experience from pre-discharge to bridging to post-discharge (for an exception see [15].

At the pre-discharge stage, the patient is in the hospital, diagnosed, and treatments are determined [11,14,16,17,18]. At the bridging stage, the patient is about to be discharged home, is educated about their condition, and is provided instructions regarding how to care for themselves once returning home [12,16,19,20,21,22,23]. At the post-discharge stage, the patient has returned home and is attempting to recover after their hospital stay [1,6,8,24].

Much of the research on factors affecting recovery has focused on the post-discharge stage, with attention given to one of three areas: personal characteristics, social supports, and the environment. Personal characteristics have been the focus of studies using large data sets and include the patient’s severity of illness, existing comorbidities, obesity, age, income, and insurance status [6,8,9,25,26,27]. Social characteristics have often received attention from qualitative researchers and have focused on social supports that can help or hinder recovery. This includes caregivers and their assistance with activities of daily living (ADL) such as eating, bathing, and dressing and instrumental activities of daily living (IADLs) including obtaining groceries, transportation, and using the telephone [6,11,27,28,29,30]. The patient’s environment has received the least attention and includes the patients’ living, housing, and neighborhood conditions affecting their recovery [1,18,24,31,32,33,34,35,36].

Currently, CMS [15] is seeking additional studies that can help further identify and clarify factors affecting UHRs. In this regard, (Shashikumar, et al. [7], p. 376) have emphasized the need for further identification of factors noting: “These include patient issues beyond a hospital’s control, such as financial and geographic barriers to primary care follow-up, health literacy, social support, and the ability to afford and manage medications and lifestyle modifications” (see also [6]). Our study has two primary aims focused on these needs: (1) to identify factors perceived by patients to influence their recovery during at least one of the three stages of the hospital to home transition and (2) to identify factors perceived by patients as important across all three stages of the transition.

## 2. Methods

### 2.1. Design

To accomplish our aims, we chose to use a qualitative approach that would give us insights into the participant’s experiences at each stage of the transition from hospital to home. This required that we develop a means of recruiting participants who had recently been discharged from the hospital (as described below). Next, we developed questions that inquired about the participant’s experiences during each stage of the transition from hospital to home. Each question was designed to be followed-up with additional questions based on the participant’s initial response. After developing the questionnaire and identifying participants, the design called for us to meet the participants wherever was most convenient for them. Consequently, most interviews occurred in the participants’ homes. Our goal was to interview roughly 60 patients since this would expend all the existing funds available for the research and would provide the necessary information to address our study aims. We used open-ended readings and the Dedoose qualitative software package version 9 to analyze the data and identify themes.

### 2.2. Procedures for Patient Recruitment and Data Collection

The Principal Investigators (PIs) partnered with a local regional hospital to conduct the study. A variety of recruitment methods were tried in order to obtain patient participation while protecting patient privacy. The most effective recruitment method was a convenience sample provided by the hospital’s Research Director. More specifically, the Research Director identified all the hospital patients who were discharged to their homes over an approximately 10-day period (roughly 130 people). We then sent each of these patients a letter from the hospital (in both English and Spanish) inviting them to participate in the study and offering a $50 gift card. If interested, the patient was instructed to contact the Principal Investigator (PI) by either telephone, email, or by returning a stamped, addressed envelope included with the letter of invitation. The first-author subsequently telephoned the 20 people who initially expressed an interest regardless of their cause for hospitalization (i.e., all-cause hospitalizations within roughly 30 days of hospital discharge). Of these, 15 agreed to be interviewed. This recruitment procedure was repeated three times over eight months between July; 2019 and February; 2020 and 62 patients were interviewed. While the data collected are roughly five years old, they are still very relevant to the on-going problem of UHRs with little change reported in the percentage of patients experiencing UHRs and on-going efforts to identify causes and solutions [37].

All interviews were conducted by the first author in English and averaged 75 min. During the interviews, the first author was joined by either one of the other PIs or a research assistant and both the first author and second researcher manually recorded the participants’ responses. We chose not to electronically record the interviews in order to obtain candid and honest answers which we believe subsequently increased the reliability of the information provided. More specifically, we feared that participants would not always be forthcoming about their hospital experiences and level of caregiver assistance knowing there was a potential for their recorded statements to be shared with hospital staff and caregivers. Further, we asked probing questions to be sure that we understood what the participant was describing which enhanced the validity of their responses. Interviews occurred in the patients’ homes except for four who chose to be interviewed in other places including a hospital reception area, university office, a park, and a motel (two of these were homeless individuals in temporary living quarters provided at discharge). The questionnaire was translated into Spanish and a Spanish translator was available to conduct interviews but no Spanish-only speaking patients volunteered to participate. Internal Review Board (IRB) approval was obtained from the local University and all proper procedures for data collection were followed to protect the privacy of the participants as required by the Health Insurance Portability and Accountability Act (HIPAA).

### 2.3. Description of the Regional Hospital (RH) and Denton County

Most of the patients discharged from RH lived in Denton County, located roughly 30–40 miles north of the Cities of Dallas and Fort Worth, Texas. RH has achieved “Magnet” status which singles it out as meeting a variety of high standards. One of these is supporting health research. RH is a for-profit hospital and contracts out its physician services. Denton County is one of the fastest growing areas in the United States with 378 census block-groups [38]. Denton County housed roughly 861,690 residents in 2020 (census bureau estimate) and included 878 square miles. Census bureau estimates for 2019 show: White = 58%, Hispanic = 20%, Black = 11%, and Asian = 10%. About 93% was urban and 7% rural. The median age was 35 with 11% aged 65 or older. Eight percent of the population was in poverty, 13% under age 65 were without health insurance, and the median family income was $83,000. About 93% of the population had a high school education and 51% were female [38].

### 2.4. Interview Protocol

The purpose of the face-to-face interviews was to identify themes at the pre-discharge, bridging, and post-discharge stages that participants believed affected their ability to recover, as well as to identify those themes influential across multiple stages. The interview protocol included questions that focused on their experiences at each stage, on their success at recovery, and on personal factors, including their income, age, gender, and height and weight in order to measure the participants’ body-mass index (BMI; a rough estimate often used to measure obesity when more exact data are unavailable). All responses to questions regarding health and recovery were followed-up with one or more additional questions to obtain more in-depth information and clarity. Questions included: “Do you feel you were physically ready to come home from the hospital?” “Do you feel the doctors and nurses gave you all the information you needed to get better?” “Did you understand all of the instructions provided or were the instructions not completely clear?” “What non-medical things have helped you to get better? That is, things that have helped you but were not provided by a medical person”. “When thinking about your recovery, what has made it most difficult for you to get better?” “What has helped you the most to recover since getting home from the hospital?”. For those who experienced an UHR, we asked an additional question: “What could have been done differently to avoid you having to go back to the hospital after being sent home the first time?”.

### 2.5. Qualitative Analytic Strategy

**Figure 1:**
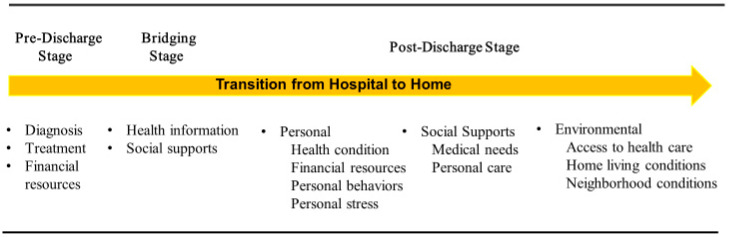
Themes and Sub-Themes Associated with Stages of Recovery During the Hospital to Home Transition.

We used a grounded theory approach to systematically analyze the data to identify themes important to patient recovery and we subsequently developed a working model displaying factors of importance to the hospital to home transition (Figure 1). To accomplish this, the first author began with an open-ended reading of the information provided by the participants. The purpose of the readings was to identify themes at the pre-discharge, bridging, and post-discharge stages that were perceived as influencing the participant’s ability to recover. To help identify themes, the experiences reported by those participants who experienced difficulty recovering (e.g., experienced an UHR) were compared to those who reported having no or little difficulty recovering [39]. In addition, a co-author coded each participant’s specific responses with the assistance of the Dedoose qualitative software. Codes were then aggregated based on their similarity so that broader themes and sub-themes were created [39,40,41]. After identifying themes at each stage, comparisons were made between stages to identify any themes that were influential at multiple stages or across the entire transition. The themes identified are described below [39,42].

## 3. Results: Factors Reported to Affect the Ability to Recover During the Hospital to Home Transition

Table 1 provides general characteristics of the 62 participants. Their average age was 62 with 50 percent female. Eighty-eight percent (88%) were white and 69% were married or living with a significant other. Sixteen percent (16%) had a high school degree, GED, or less education, while 36% had some college and 48% had a two-year college degree or higher. Regarding insurance, 8% had none, 16% had Medicaid, 52% had Medicare, and 65% had private health insurance (some participants had more than one type of insurance). Primary pathologies varied widely with respiratory issues making up 21%, heart problems 8%, pulmonary 13%, other serious conditions 42%, and less serious conditions 16%. The average stay in the hospital prior to their initial discharge was 4.8 days, with 19% of the participants experiencing an UHR. Provided below are the themes or factors identified by participants as important to their ability to recover during the hospital to home transition. 

### 3.1. Pre-Discharge Themes Identified

Three pre-discharge themes were identified as factors believed to influence the participant’s ability to recover (Figure 1). These included participant reports of incomplete medical diagnoses, inappropriate treatment, and lack of financial resources, with the three described as interrelated. For example, Participant 45, a 40-year-old man, believed his medical diagnoses and prescribed treatments were influenced by his inability to pay for the health services provided. He reported being admitted into the hospital with a rapid heartbeat and fainting. He believed he was subsequently “rushed” out of the hospital without a thorough examination and treatment because he had no money or insurance. After his discharge and subsequent UHR, he was transferred to a large public hospital that receives federal funds to treat indigent patients. He said that doctors there examined him more thoroughly and found fluid on the heart, circulation problems, and problems with his pancreas. Once he was discharged, they continued to treat him.

Another example of perceived inappropriate diagnosis and treatment was provided by Participant 62, a 59-year-old female. She complained that, during her initial hospital stay, the hospitalist prescribed steroids for her spinal pain and sent her home without consulting with her surgeon who was recommending surgery. She subsequently did not recover, experienced an UHR, and believed her eventual diagnosis and treatment could have been correctly determined at her first hospital stay if the hospitalist had consulted with the spinal surgeon. Unlike these two cases, participants who reported recovering successfully, did not have such complaints. Instead, these participants generally reported having health insurance or other means of paying for their health services and expressed thanks to the medical staff for doing an excellent job diagnosing and treating them while they were in the hospital.

### 3.2. Bridging Themes Identified

Two interrelated bridging themes were identified as affecting the participant’s ability to recover: amount and clarity of health information at hospital discharge (HD) and lack of social supports accompanying the patient during HD. Several participants who were having difficulty recovering believed they were not given enough information at HD to adequately care for their health issues. Participant 12 was admitted to the hospital for cellulitis. She complained of not being instructed to avoid soaking her leg in water. Without this information, she returned home and did soak her leg regularly since it helped reduce her pain. However, she learned, after her UHR, that soaking her leg was a contributing factor to her lack of healing and subsequent readmission.

Some of the participants, who lacked social supports during the bridging stage, reported difficulties understanding the information provided. Consequently, once returning home, they were unsure of how to care for their health issues. These participants explained that, during the HD process, they were feeling the effects of various medications that had been given to them and/or were still in shock from the traumatic experience of being hospitalized and so were not thinking clearly. As a result, they had difficulty understanding the instructions provided. Participant 35, a 61-year-old woman, reported that she had no help from family or friends to understand the instructions given and reported the information she received was confusing, including how to use a neck brace she was instructed to wear. She also had no help driving home and, as she attempted to drive the 40 miles by herself wearing her neck brace, her neck became very painful. As a result, she immediately went to the RH emergency room which was located near her home and was readmitted (though it was not the hospital where she was originally treated).

Participants, who reported recovering successfully, often noted the value of having social support at the bridging stage while receiving medical instructions. For example, Participant 9, a 51-year-old woman, explained that she shattered her ankle. During the HD process she was “not in a good state of mind” as she was still experiencing some shock from her accident. She noted the good fortune of “having someone there to hear the instructions” during her discharge.

### 3.3. Post-Discharge Themes Identified

We identified three post-discharge themes and these mirrored those identified by previous research: personal characteristics, social supports, and the environment. Further, within each of these, we identified sub-themes (Figure 1). When considering the personal characteristics theme, four sub-themes were identified: health condition, financial resources, personal behaviors, and personal stress. As reported in previous studies, it was no surprise that the existence of severe health conditions was clearly associated with one’s ability to recover. Participants with severe conditions were typically found to have co-morbidities and in many cases were obese. For example, Participant 4 was an 89-year-old obese woman who was admitted to the hospital for bronchitis and congestive heart failure. Upon discharge, her recovery plan included prescribed exercise. However, she reported being unable to follow the medical instructions and routinely missed medical appointments due to her difficulty moving and walking.

A second personal sub-theme was lack of financial resources. This was found to be associated with many of the participants who experienced an UHR and described as preventing them from doing a variety of activities including: affording medications, eating healthy food, living in a healthy environment, securing daily needs (e.g., rent, food), obtaining transportation to medical appointments, and finding health care professionals willing to see them, including medical doctors (MDs) and physical therapists (PTs). For example, Participant 32 was a 57-year-old man admitted to the hospital with vascular disease. While he was covered by Medicare Disability Insurance and Medicaid, he reported not having enough money to follow the instructions given. He also reported having difficulty getting to medical appointments. On the other hand, those participants who appeared on the road to recovery, had the financial resources for recovery or had family or friends who provided the funds required. Participant 01, a 61-year-old man, was one of these cases. He noted that he could not afford a hip replacement but his girlfriend and family members provided him with the funds necessary to have the procedure.

Closely tied to a lack of financial resources were reports of insufficient or no health insurance contributing to the inability to obtain needed health services. For example, Participant 13, a 67-year-old man, had Medicare and a low income. Following his discharge from the hospital, he was instructed to see a pulmonologist to help him with his breathing issues but reported he could not afford it even though he did receive Medicare. He further reported that he had difficulty finding an MD who would accept Medicare and could not afford all the medications he had been prescribed.

A third personal sub-theme was personal behaviors, including the use of tobacco products and not following medical advice. Participants 32 and 45 both reported that they had been smoking for decades and continued to smoke after HD, which was against medical advice. It is reasonable to suspect that their reported difficulty in recovery was due at least in part to their choice of ignoring medical advice and continuing to smoke.

A final personal sub-theme identified was personal stress. Those who reported difficulty recovering typically expressed feelings of high stress because of the uncertainty surrounding whether they would recover. On the other hand, those who felt they were clearly on the road to recovery reported feeling stressed but believed they were keeping it under control. Other factors reported to cause high stress included a lack of financial resources to pay for health needs, such as follow-up medical appointments and prescribed medicines, and lack of social support to help with daily ADL and IADL needs (e.g., making medical appointments, providing transportation).

The second post discharge theme, social supports, was found to have two sub-themes: support for medical needs and support for personal care needs. Some participants, who reported difficulty recovering, also reported lack of help with following medical instructions on a daily basis. Some also reported problems achieving personal care needs including lack of help with ADLs and IADLs. Participant 21 was a 61-year-old obese woman who lived by herself in a small trailer with very little income and minimal social supports. She reported that, once being discharged from the hospital, she had insufficient help obtaining and taking her medications and maintaining her personal hygiene which meant she bathed less than once a week. Three weeks after her discharge, she developed pneumonia and kidney failure, resulting in an UHR. On the other hand, participants who reported having various types of assistance, such as spouses helping them get dressed and neighbors providing meals, were more likely to also report successfully recovering.

The third post-discharge theme, environment, was found to have three sub-themes: access to health care, home living conditions and neighborhood conditions. Many of the participants who reported difficulty recovering noted health access problems, such as obtaining transportation to see specialists, and attributed these problems in part to living far from the specialists they needed to see. Participant 2, a 58-year-old woman, reported that she needed to be seen periodically by an ears, nose and throat specialist. The closest one approved by her insurance plan was located in Dallas over 40 miles away. She had difficulty obtaining transportation, missed some of her appointments, and believed this difficulty inhibited her ability to recover.

Some of those reporting difficulties recovering believed it was, in part, due to their home living conditions, including exposure to second-hand smoke, unhygienic surroundings, and mold. Participant 13, a 67-year-old man, had experienced multiple UHRs due to chronic obstructive pulmonary disease (COPD). He reported that he had stopped smoking for several years but his wife still smoked in their home. While she had restricted her smoking to only one room, he reported the second-hand smoke negatively affected his breathing. Participant 20 was a 72-year-old woman living in public supported housing. She reported being readmitted to the hospital multiple times over the past several months with doctors unable to determine what was causing her symptoms. She further reported that she thought her illness might be exacerbated by a large amount of mold she had discovered in her window air conditioner. She had notified the apartment management several months earlier but they had not attempted to remove it or respond to her. She explained that her health would improve while in the hospital but once returning home would progressively get worse and eventually she would experience another UHR. At the time of the interview, she had not notified the doctors about the mold. We subsequently obtained the participant’s permission to measure the air quality in her home and found it to be extremely unhealthy with 65 particles per 0.01 cf. (good air quality is reflected by a measurement of 0–12 particles per 0.01 cf.).

Unhealthy and unsafe conditions within the neighborhood were also described by some as inhibiting their ability to recover. Participant 2, who had been hospitalized with pneumonia, was instructed to get regular exercise once returning home. Consequently, she went for walks every morning in spite of a nearby wheat field being harvested. She reported that, in hindsight, she realized the wheat particles, along with exhaust from cars and local gas wells, aggravated her respiratory problems and inhibited her ability to recover. On the other hand, participants who reported to be successfully recovering, tended to report healthy, safe, and, for some participants, enjoyable and relaxing neighborhoods.

When considering themes found at multiple stages or across the entire transition from hospital to home, four interrelated themes were identified including financial resources, access to health care, social supports and personal stress. These were perhaps the most often cited by participants who experienced an UHR when they were asked: “What could have been done differently to avoid you having to go back to the hospital after being sent home the first time?” These are discussed further below.

## 4. Discussion: Factors Influencing Recovery Across the Hospital to Home Transition

The inability to recover after a hospital stay is a serious problem, resulting in readmitted patients who experience an inconvenience at best and death at worst, as well as costing the health care system billions of dollars [1]. A major step in addressing the high number of UHRs is to identify the factors associated with them. Our aims were to identify the factors associated with UHRs at each stage in the transition from hospital to home, as well as to identify factors that influence UHRs across this transition.

To accomplish our aims, we interviewed 62 participants recently discharged from the hospital. As a result, we identified themes associated with recovery at each stage. These are discussed above and presented in Figure 1. From these themes, we identified four that appeared to be influential at multiple stages of the transition: financial resources, access to health care, social supports, and personal stress. Financial resources were reported to be influential at the pre-discharge stage and highly interrelated with access to health services. Some of those who reported difficulties recovering believed the lack of financial resources reduced their access to adequate diagnoses and treatments while in the hospital. These participants reported feeling “rushed” and “pushed” to be quickly discharged because of their inability to pay. This complaint has been reported in previous research [11]. Once returning home, some participants who lacked financial resources reported continuing to have difficulty accessing all the health services needed, as well as the ability to have a healthy living environment and lifestyle. This included access to medical treatments, pharmaceuticals, healthy foods, transportation, and healthy living conditions. On the other hand, those who believed they were recovering successfully typically reported having adequate financial resources and access to health services. While few studies were found to consider the impacts of financial resources throughout the hospital to home transition, many studies have highlighted its importance at varying stages [20,27].

The availability of social supports was reported to assist recovery beginning at the bridging stage and into post-discharge. On the other hand, those who lacked social supports believed this contributed to their difficulty recovering. This included difficulties obtaining and understanding the medical instructions and for at least one participant obtaining transportation home from the hospital. There is relatively little information provided by previous studies on the bridging stage of the transition. Once returning home, participants reported difficulty obtaining help with some of the ADLs and IADLs needed, such as maintaining their personal hygiene, preparing meals, cleaning their home, and making and attending medical appointments. Those reporting to be recovering successfully noted a variety of ways that their social supports assisted them, from taking a shower to providing a meal to providing transportation to medical appointments. This supports previous findings focused on recovery at home [30].

When considering personal stress, past research has shown the negative health effects it can have when continually experienced over a long period of time [43,44,45]. However, little research has been found to consider it in the context of the hospital to home transition. This theme was found at all three stages of the transition, though most notable at the post-discharge stage. At pre-discharge, some reported high levels of stress as they considered their health condition, tried to figure out how they would pay for their hospital stay, or felt stress knowing that they could not pay. At the bridging stage, some reported high stress as they attempted to understand the instructions being provided, particularly if they lacked social supports to assist them. At the post-discharge stage, high levels of stress were reported when access to health care could not be afforded, personal care needs could not be met, and living conditions were not healthy. Perhaps most stressful for those who experienced an UHR was not knowing whether they would experience another UHR. Those who believed they were recovering successfully noted feelings of stress but also the ability to keep it under control, particularly if they had the help of social supports and the financial resources needed during recovery.

## 5. Limitations

While our qualitative study does provide information that helps identify factors associated with one’s ability to recover during the hospital to home transition, the conclusions reached are not generalizable due to the small sample size and the participants coming from just one area within Texas. In addition, the 62 participants were treated and discharged from the same hospital. It is possible there were unique characteristics of the hospital that influenced our findings. The inclusion of additional hospitals would have helped to overcome this shortcoming. Further, while we used a convenience sample, if there was no sampling bias, the characteristics of the 62 participants would be similar to those patients discharged from the hospital who chose not to participate in the study. Unfortunately, we were unable to obtain the characteristics of those who chose not to participate, limiting our ability to assess sampling bias in this regard. Further, we did not have enough participants to consider different experiences by age, race, or health condition due to funding limitations. While this would have been valuable information, we do feel that what was uncovered lends itself to identifying important factors influencing the participants’ ability to recover after their hospital stay. The study relies on the participants to correctly remember their pre-discharge, bridging, and post-discharge experiences. Ideally, we would have also interviewed medical and other staff to verify participant accounts and reviewed the patients’ hospital discharge summaries for verification and completeness. However, this was beyond the time and financial resources available.

## 6. Conclusions

The inability to recover after a hospital stay is a serious problem in the U.S., with roughly 18% of discharged patients experiencing an unplanned readmission within 30 days. To reduce this problem, a first-step is to determine the factors associated with recovery during the hospital to home transition, including factors at the pre-discharge, bridging, and post-discharge stages. Our study aimed to (1) identify factors perceived by patients to influence their recovery during at least one of the three stages of the hospital to home transition and (2) identify factors perceived by patients as important across all three stages of the transition. Previous research has focused on three stages with most studies concentrating on only one of the three and many others lacking in-depth information from the participant’s perspective. Our research has taken a more wholistic approach by obtaining the participants’ perceptions of their experiences through all three stages. Each stage was reported to have factors that influenced one’s ability to recover. At the pre-discharge stage adequate diagnoses and treatments, along with a lack of financial resources, were viewed as affecting recovery. At the bridging stage, the inability to obtain complete health information and a lack of social supports were perceived to inhibit recovery. At the post-discharge stage personal, social, and environmental factors were believed to affect recovery. When considering factors that were perceived to influence recovery across multiple stages, four were identified: financial resources, access to health services, social supports, and personal stress.

## Figures and Tables

**Table 1:** Characteristics of the 62 patients interviewed.

Characteristics of Participants	
Average age	61.9
% Female	50.0
% White	87.9
% Married or living w/significant other	69.2
Education	
% less than high school	6.6
% high school/GED	9.8
% some college	36.1
% 2 year or more college degree	47.6
* Available insurance	
% None	6.5
% Medicare	51.6
% Medicaid	16.1
% Private insurance	64.5
Primary pathology	
% Respiratory	21.0
% Heart	8.1
% Pulmonary	12.9
% Other serious condition	41.9
% Less serious condition	16.1
Average # of days in hospital	
prior to initial discharge	4.8
% readmitted within 30 days	19.4

* Percentages don’t sum to 100 percent because some participants had both Medicare and private insurance.

## Data Availability

The data that support the findings of this study are available for academic research purposes from the corresponding author upon request.

## Public Involvement Statement

There was no public involvement in any aspect of this research.

## Guidelines and Standards Statement

This manuscript was drafted against the Standards for Reporting Qualitative Research for qualitative research [46].
